# Pharmacology through Play: using Lego® to revise core concepts for undergraduates

**DOI:** 10.15694/mep.2019.000201.1

**Published:** 2019-11-14

**Authors:** Brian P Kirby, Teresa Pawlikowska

**Affiliations:** 1 School of Pharmacy and Biomolecular Sciences; 2 Health Professions Education Centre

**Keywords:** Pharmacology, Lego®, Constructivist theory, Play, Medical Education

## Abstract

This article was migrated. The article was marked as recommended.

**Background:** Pharmacology, while critical knowledge for healthcare professionals, is often viewed by students as dry and difficult to understand. We sought to examine the student acceptability of a Lego®-based learning session, in an attempt to improve pharmacology learning.

**Methods:** In line with constructivist theories, students were facilitated to build, in small groups, their own Lego® shape to represent an area of core pharmacology and to use this to explain the concept to other students (e.g. agonist-receptor interactions). The validated Course Experience Questionnaire (CEQ) was used to gauge students’ ideas on the session. Multiple choice questions were used before and after the session to evaluate knowledge.

**Results:** Most students were positive regarding the session, finding it enjoyable, relevant for their learning and even recommending it be used to explore more complex areas of pharmacology. In addition, there was a significant increase in the MCQ scores following the session.

**Conclusions:** This study used constructivist theory to develop a novel teaching intervention to create a more student-centred, active learning environment. This effective low-cost method could be applied to other teaching programmes to enhance student learning.

## Introduction

Pharmacology is a core component of any health sciences curriculum, in particular, pharmacy and medicine degrees. There is an ever expanding body of pharmacological agents falling within the remit of healthcare professionals, particularly pharmacists as recognised medication experts. Without a good grasp of the underlying basic principles of pharmacology, students, and ultimately practicing healthcare professionals, will find it more difficult to integrate this new information and use it to make informed decisions about drug use and therapeutic regimes. It is important therefore to ensure these generic, transferable skills are well taught and understood by students.

Because of the increasing volume of information in the field of pharmacology, there has often been considerable focus on content rather than attending to the learning process, resulting in information overload ( Achike and Ogle, 2000). In response, attempts have been made to develop more interactive methods to teach pharmacology in order to promote more active learning and a deeper understanding ( Sharma *et al*., 2004; Zgheib, Simaan and Sabra, 2010). Hence, the teaching of pharmacology has become more learner-centred, with the traditional lecture-based teaching delivered in conjunction with more novel and interactive methods. These mixed approaches are important to ensure students remain engaged with their learning.

One of the mainstays of pharmacology education has always been so-called ‘wet practicals’ involving isolated tissue preparations, whereby students apply their knowledge in a hands-on approach to practical pharmacology. These are particularly useful in demonstrating the basic principles of drug & receptor interaction. However, given the increased expense and ethical issues surrounding such practical laboratory classes, alternatives have to be found. Computer simulations have been used to replace the isolated tissue preparation practical classes ( Aarons *et al*., 1988; Coleman *et al*., 1995,; Dewhurst, Hughes and Williams, 1996; Kuruvilla *et al*., 2001). However, while a useful alternative, which promotes independent student learning due to the students’ need to organise their own study time effectively ( Dewhurst, Macleod and Norris, 2000), the learning could be viewed as being less active as there is no ‘hands-on’ component. It is important, therefore, to develop new approaches of teaching pharmacology that incorporate active learning tasks and thereby allow students to form and implement their learning more easily.

Numerous studies have shown that allowing students to explore and construct objects to demonstrate their understanding leads to deeper learning of the concepts. Hobby kits and toys, such as Meccano®, have been used in the teaching of engineering and more broadly in the teaching of STEM subjects ( Parkin, 2002; Das, Yost and Krishnan, 2010). In pharmacology, to illustrate different concepts, novel methods have been used such as simulated role play ( Richardson and Maddock, 2013), the use of crosswords ( Gaikwad and Tankhiwale, 2012) and even pharmacology songs ( MacDonald and Saarti, 2006). Linked with the use of objects, and stemming from a consideration of how young children learn, play has been shown to enhance learning and, consequently, life-long learning through life-long play has been advocated ( Farne, 2005). This so-called “Pedagogy of Play” has its basis in constructivist theories and could be a useful active learning technique to apply in the pharmacology class. Furthermore, combining this with group work and peer-demonstration/teaching, which have been widely demonstrated to be effective teaching methods ( Krych *et al*., 2005; Letassy *et al*., 2008; Beatty *et al*., 2009), should lead to an active and supportive learning environment which will further the students’ understanding and application of the basic principles of pharmacology.

The theory of constructivism, as articulated by Piaget ( Piaget, 1971), is concerned with active learning whereby students create and further their own understanding through their own perceptions and experiences. The overall aim of this study was to introduce and evaluate a novel teaching method based on constructivism and using Lego® for play, to complement the existing teaching of the basic principles of pharmacology and to aid pharmacy student learning. The intervention used elements of play, teamwork and peer-teaching to revise core concepts in pharmacology.

## Methods

We hypothesised that students would benefit from a constructivist educational intervention through increased understanding and ability to apply their knowledge of the basic principles of pharmacology.

The objectives of this study were framed according to the principles of educational evaluation developed by Kirkpatrick ( Kirkpatrick and Kirkpatrick, 2007; Kirkpatrick and Kirkpatrick, 2016).

1. Reaction/Perception: To examine the feasibility of using Lego® to revise core pharmacology topics.
2. Learning/Outcome: To determine the acceptability of the use of Lego® as a teaching tool for students and the impact on learning.

### Sample

Students were recruited from second and third year Pharmacy classes. For normal teaching and academic purposes, these students are routinely allocated to two groups (A&B).

**Figure 1.**
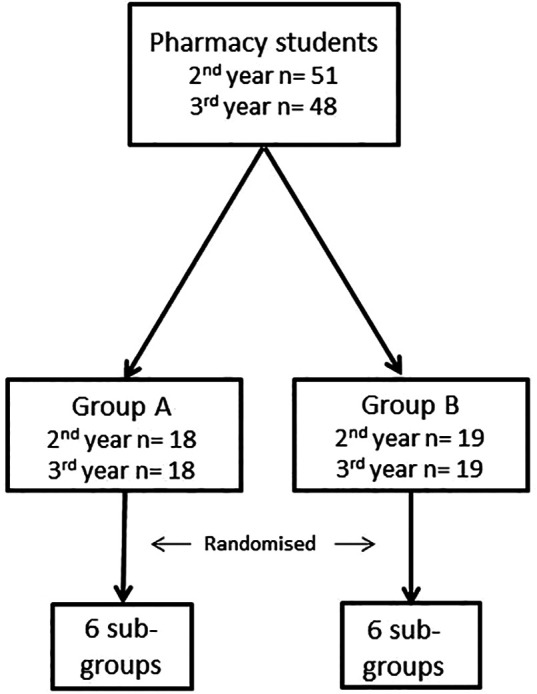
Illustration of the sampling and anonymization of study participants. Students from 2 ^nd^ and 3 ^rd^ year Pharmacy, previously split into groups A and B, were subsequently randomised in to one of 6 sub-groups.

While participation in this novel optional activity was voluntary, it was scheduled as part of the students’ normal timetable. Volunteers in each group, from both the second and third years, on attending the teaching session, were randomised and anonymised through allocation of a study number (drawn ‘out of a hat’) ( Figure 1). The intervention was conducted in two sittings, with the second immediately following the first.

### The intervention

The focus of the session was to help student learning by providing the opportunity to recall and re-imagine their understanding of the basic concepts of pharmacology (see below). Through group work, and subsequent peer-led teaching, the students refined and shared their knowledge of core pharmacology concepts.

Students answered a 30-question multiple choice (MCQ) assessment on the core principles of pharmacology, selected from an existing MCQ bank. This allowed the establishment of baseline knowledge prior to the learning session which then had the following format:

• Overview of structure, learning outcomes and approaches to the session
• Directed play time focussed on one assigned area of pharmacology (agonist-receptor interactions (ionotropic receptors); agonist-receptor interactions (metabotropic receptors); competitive antagonists; non-competitive antagonists; partial agonists; inverse agonists). Plastic building blocks (Lego®), were used during the session to allow the students to work together to create a Lego® construction that helped them to understand and describe their assigned area of pharmacology.
• Presentation to peers of each student groups’ understanding of their assigned area of pharmacology with questions asked by other students and the facilitator.
• Students then sat the same MCQ at the conclusion of the session. The purpose of the MCQ was to gauge, through analysis of individual’s results, whether or not there was an improvement in MCQ scores as a result of the session. After the session, students were invited to complete an online questionnaire, (5-point Likert scale and free-text answers), to gauge their perception and the acceptability of the teaching intervention.

### Ethical considerations

All participants were provided information regarding the session, via an independent gatekeeper, and subsequently, gave informed consent before participating as required by the institution’s Research Ethics Committee and the Declaration of Helsinki (1964).

### Data analysis

A paired t-test was used to compare individual students’ scores in the MCQ assessment before and after the intervention ( Barry, O’Sullivan and McCarthy, 2015). This pragmatic approach, with a short timespan around the timing of the MCQs and the use of the same instrument, was adopted to identify change as a result of the intervention.

The validated Course Experience Questionnaire (CEQ) was used to examine students’ acceptance of the novel method and a self-assessment of their own learning as a result of the session, it included negatively scored questions ( Ramsden, 1991; Wilson, Lizzio and Ramsden, 1997). Most questions were scored on a 5-point Likert scale (1=strongly agree to 5=strongly disagree). Students were asked for free-text responses to three questions concerning; what worked well in this session, suggested improvements, and perceived value.

## Results/Analysis

Participation was high (73% and 77% of the total 2 ^nd^ and 3 ^rd^ year students respectively) and all students engaged in the Lego® play with considerable interaction within the groups. The two cohorts of students were broadly similar in terms of demographics. The entire second year class had a mean age (± standard deviation) of 21.1 ± 2.7 with 39% male students and EU:non:EU ratio of 5:1 compared to third year 21.5 ± 2.3, 30% male and 2.64:1 (EU:non-EU). These proportions were not statistically different between the two groups (p=0.33 and p=0.19; gender and nationality respectively).

### Lego® Constructions

**Figure 2.**
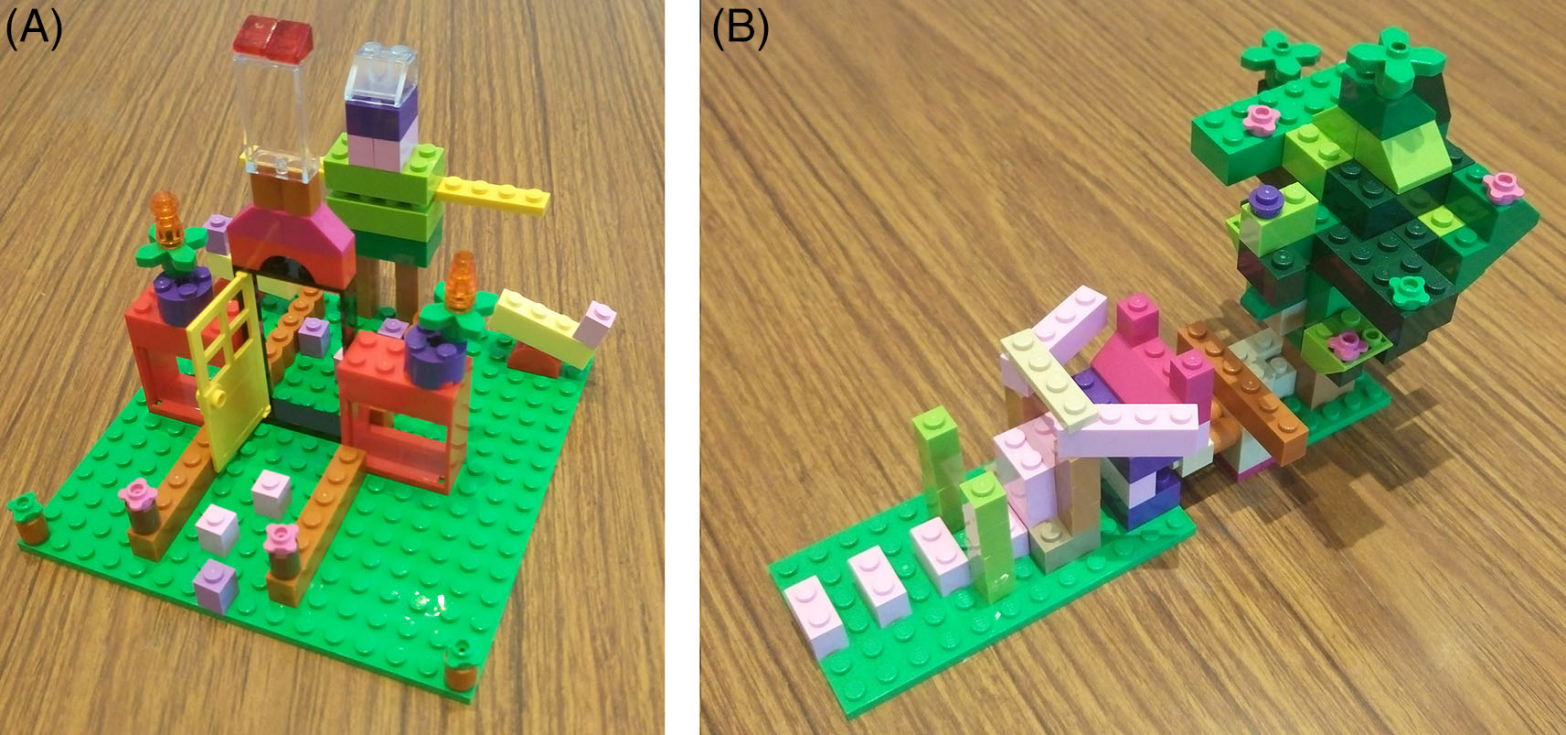
Photographs of sample Lego® models constructed by students to explain basic pharmacology concepts during the play session. A) Ionotropic receptors B) Inverse agonists.

Each group of students made a Lego® creation to represent their assigned area of pharmacology. While some created models similar to illustrations found in pharmacology texts, most groups created very different objects that explained the concept in an unusual but understandable and memorable manner. Figure 2a & b shows the creations of two groups, which were used to explain the action of agonists on ionotropic receptors and inverse agonists respectively. These models were novel and based on the students’ own re-imagining of the function of receptors.

### Course Experience Questionnaire (CEQ)

Student response rate was 43% (32 of 74 who attended the session). This was in line with other surveys conducted within the institution and also in line with other published online surveys of teaching (average 33%) ( Nulty, 2008). Responses to the set questions ( Table 1) show the Lego© session was very positively received with the majority of students agreeing or strongly agreeing with most statements. Students appreciated the style of the session, particularly the interactive student/lecturer style (84% agreed or strongly agreed), and reported that the session aided their understanding of the basic concepts of pharmacology (76% agreed or strongly agreed; Table 1).

**Table 1.** Selected CEQ responses provided by participants on completion of the play session.

	SA	A	N	D	SD
The session was interactive and this enhanced my learning (n=31)	**29%**	**55%**	**10%**	**6%**	**0**
The time and workload for the session was adequate (n=32)	**28%**	**50%**	**16%**	**3%**	**3%**
The teaching session enhanced my understanding of the basic concepts of drug receptor interactions (n=32)	**28%**	**47%**	**19%**	**6%**	**0**
As a result of this session, I feel more confident about my knowledge of the basic pharmacology principles (n=32)	**12.5%**	**50%**	**25%**	**12.5%**	**0**

SA Strongly Agree; A Agree; N Neutral; D Disagree; SD Strongly Disagree

In their free-text responses when asked what worked well, students were extremely positive, commenting that ‘the unusual methods used in this session made it very engaging and for the majority of the time it didn’t even feel as though we were trying to understand difficult concepts’(student #7) and ‘the Legos were fun to work with and the fact that we had to think of how we were going to represent our knowledge through them is what I think helped us remember the concepts better’(student #29).

Regarding improvements, there were useful suggestions around sequencing, ensuring that this intervention is timetabled closer to the teaching of the core concepts of pharmacology (these comments were mainly from the Year 3 Pharmacy students). Students found the session useful and applicable to other areas commenting that they would like ‘more time with the Legos’ and it could cover ‘further topics not just agonists and antagonists’(students #10 and #12, respectively).

Finally, the answer to the third question (Do you think this session would fit well with other parts of the Pharmacy course and be of value to Pharmacy students?) was a resounding ‘yes’. Students thought it could be ‘an easier way to learn the topics and solidify the key concepts’ (student #27) and that ‘it could provide a practical, hands-on method of learning to compliment the abstract theoretical aspects of our course’(student #18). One student voiced a contradictory opinion the he/she would ‘just get frustrated with the small fiddly Lego pieces but perhaps for others it might be beneficial but not for me’ (student #25) but this contrasted with another student who stated that he/she is ‘a kinaesthetic learner, [and] this session helped [him/her] remember basic concepts from recalling the shape of the Legos’ (student #2).

The other beneficial element of the session that was apparent from the student feedback was around peer-learning. Students appreciated both peer learning and interactive learning and that the simple explanations used helped learning as ‘other peoples’ understanding often helps as lecturers can sometimes complicate an example whereas peers tend to simplify it right down so it’s easier to remember’ (student #19).

### Pre-, post-MCQ assessment

**Figure 3.**
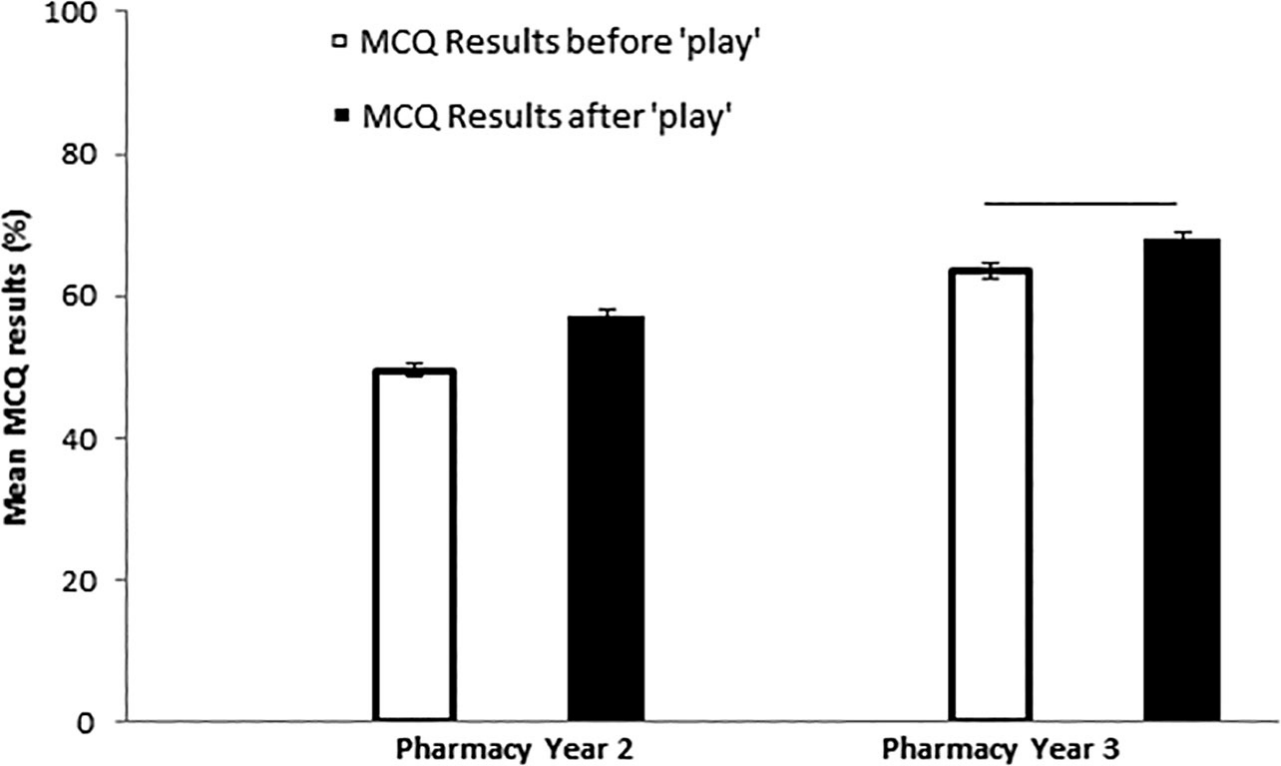
Graph illustrating the improvement in MCQ scores following the Lego ® play session for both 2nd and 3rd year pharmacy students. Mean±SEM. ** p<0.01; *** p<0.001 (paired t-test)

There was a significant improvement in MCQ scores following the Lego® play intervention (p<0.001, Figure 3) and Year 2 students showed a greater improvement compared to the Year 3 students (7.6% (p<0.001); 4.4% (p<0.01)). This was consistent with the questionnaire responses and free text in which the students reported benefit to their learning ( Table 1 and above).

### Costs

Two “classic large creative brick” Lego® boxes were used for the session, costing €40 each. This initial, once-off outlay could continue to be used for future sessions. In addition, employed staff costs can be estimated and would represent a recurring cost. To estimate this, the pay rate for individual external lecturers was used, meaning that to run this session, the estimated cost was €5.19 per student. Going forward, the only recurring costs would be those of staff and occasionally replacing the Lego®.

## Discussion

This study has demonstrated a novel approach to the revision of core concepts in pharmacology that could be utilised in any programme where pharmacology is taught. Focussing on constructivist theory and using group work and peer-teaching, we used Lego® building blocks as a teaching tool which the students clearly enjoyed and felt was applicable and beneficial to their studies. Engagement with the session was high. In addition to their perceptions, we demonstrated that the intervention had a beneficial impact improving their MCQ scores. Furthermore, this offers a cost-effective method for increasing active learning, useful for large numbers of students.

In the healthcare setting, as care models of practice evolve, healthcare professionals, and in particular pharmacists, are expected to be able to provide expert knowledge on drug action and interaction. Therefore, it is vital that the core concepts of pharmacology are effectively taught to ensure that students and qualified professionals are able to integrate and utilise all of the information that depends on them. We have demonstrated that using Lego® building blocks, to allow the students to explore and share their understanding of basic pharmacology, improved performance in an MCQ knowledge test. Furthermore, the students reported that the play session was interesting, enjoyable and supported their learning.

The need to engage students in their own learning has been recognised for some time but the move from purely lecture based teaching has been slow in pharmacology. Poor retention of knowledge from lectures is high: McKeachie showed students can, on average, only recall 70% of the material from the first 10 minutes of a lecture, dropping to 20% of the content from the final 10 minutes ( McKeachie, 1986). While there are caveats e.g. individual differences in attention or whether retention would be reflected in student notes ( Wilson and Korn, 2007), it nonetheless highlights the need to engage students in different ways to improve their attention, engagement and learning. More recent evidence has shown even lower retention, with Bligh finding students recalled less than 10% after three days ( Bligh, 2000).

A review from 2009 by Jungnickel and co-workers examined pharmacy practice and the development of future competencies with recommendations for the education of pharmacists. One highlight was the need for student- or learner-centred teaching, with the suggestion that problem-based learning (PBL) may be one method to achieve this ( Jungnickel *et al*., 2009). Certainly there is evidence that PBL helps student engagement ( Galvao *et al*., 2014) as do other learning strategies that have been widely investigated in pharmacy and other healthcare education programme. These include process-oriented guided inquiry learning (POGIL), clicker or audience response systems and team-based learning ( Beatty *et al*., 2009; Soltis *et al*., 2015; Stevens *et al*., 2017). The key feature with all of these is that they are active, rather than passive, forms of learning and all attempt to reduce the extraneous cognitive load.

Our intervention uses a combination approach to the active learning session. The first part was a hands-on, active play session allowing the students to construct a representation of their understanding. This aligns with the constructivism as the students demonstrated their own understanding with their Lego® construction but also in the way they create new understanding through their interaction with other students ( Piaget, 1951; Piaget, 1971). Similarly, our intervention aligns with the constructionist view on the development of knowledge (inspired by constructivism and developed by Papert) in that it is student centred, involves discovery learning with tangible objects that allows the students to build something to represent and further their knowledge and understanding ( Papert and Harel, 1991). It is also evident in the creation of the Lego® models as their understanding and meaning is personal to them ( Bruner and Anglin, 1973). In addition, we used elements of peer-led team learning, which has also been extensively evaluated in undergraduate science and health science programmes ( Preszler, 2009; Walpola *et al*., 2015). Peer-led teaching and team work allows students to engage with each other and to enhance their learning in a supportive environment ( Eberlein *et al*., 2008), which has been demonstrated to benefit some students, and in particular students’ critical thinking skills ( Lewis, 2011; Wilson and Varma-Nelson, 2016). The freetext comments provided by the students clearly illustrate the value they placed on the peer-led aspect of this intervention, further underlining its importance.

The decision to use Lego® was based on growing literature around the importance of play as a learning tool and also the recent use of Lego® as a management tool to explain and understand processes - Lego® Serious Play® ( Frick, Tardini and Cantoni, 2013). Play is important for learning, not just in children, but through life, as long as it is appropriate play, building on constructivist and constructionist theories ( Piaget, 1951; Papert and Harel, 1991). Lego® Serious Play® was built on these principles and has been used in business settings to allow people to, for example, understand the management structure and functioning of an office or department. Linked with this use are the theories around the mind-hand connection and haptic feedback that is received during play or construction ( Roos and Victor, 1999; PLAY, 2002; Chang and Yeh, 2015). These all contribute to the understanding and learning of the concepts. Furthermore Lego® has been used to stimulate reflective practice on teamwork skills in the third level setting ( Seidl, 2017). Other authors used Lego® Serious Play® to allow pharmacy students to explore definitions of professionalism ( Wilson and Becket, 2012). Our session used many of the same principles but applied them to a different area of learning and has further highlighted the value of a hands-on experience in science education ( Felder, 1993).

The final advantage of this technique was its cost effectiveness. All teaching establishments have embraced technology-enhanced learning (TEL), but striving to use the latest technologies has significant cost associated with them, particularly set-up costs. Indeed, to study drug receptor interactions, 3D virtual environments and 3D printing have both been used ( Richardson *et al*., 2013, Hall *et al*., 2017). Given the additional cost of human time it was surprising that more authors do not comment on cost effectiveness of novel teaching interventions. Costs and resources have hardly been explored to date although potential frameworks have been long proposed: Carpenter ( Carpenter, 1995) (personnel, standardised patients and administrative), Reznik ( Reznik *et al*., 1993) (development, production, administration and analysis), and Poenaru ( Poenaru *et al*., 1997) (costs of materials, indirect costs e.g. salaries). When it comes to technologies, the argument for cost effectiveness is usually based on the long-term use of the technology and applicability to other areas, thus mitigating costs ( Lewis, 2014). Certainly in the case of Lego® it is both cheap and applicable to different areas.

### Limitations

We used Kirkpatrick to frame our research question ( Kirkpatrick and Kirkpatrick, 2007). While there has been debate over its suitability to examine interventions in medical education ( Yardley and Dornan, 2012), we felt that it was applicable for our intervention, particularly after Kirkpatrick’s recent revision ( Kirkpatrick and Kirkpatrick, 2016).

While a crossover design with a control group may be ideal, it was not possible in this situation with an already finalised annual timetable. Furthermore, it was not feasible in the timeframe of this study to track results over a longer time span, as it is anticipated that active learning techniques have a long-term positive impact on student examination scores ( Ernst and Colthorpe, 2008). However, evidence has shown that the use of Lego®, albeit in a different context, has provided long lasting and significant learning ( Harding and D’Eon, 2001). Both of these (crossover and longitudinal tracking) should be investigated in future work and also extrapolated to examine other healthcare professions. While the students’ results showed an improvement in MCQ score, as the same set of questions was used, one cannot rule out a test-retest phenomenon, however, what is most encouraging is the student-reported responses to the use of Lego®, which was overwhelmingly positive. Finally the interpretation of the results is influenced by the fact that the students were self-selected. The session formed part of their normal teaching schedule and while the attendance reflected normal tutorial attendance levels amongst our students, it must be remembered that the students decided themselves to attend the teaching session.

## Conclusion

Students had a positive response to the introduction of the Lego® session and felt they benefitted from it through improved understanding. This was evident in the improvement in MCQ scores following the Lego® intervention. Furthermore, significant effort is going in to developing novel teaching techniques, often taking advantage of cutting-edge technology, such as simulation and other Technology Enhanced Learning approaches. This study illustrates the possibility of using Lego® as a cost-effective tool in the teaching of pharmacology, but this should not be restrictive and other areas of healthcare education could well benefit from its use as an additional student-centred learning activity, particularly due to its engagement, cost and accessibility.

## Take Home Messages

• Lego and other construction based tools are effective for teaching complex subjects to health professions students
• Pharmacy students enjoy the Lego based intervention, which improves their engagement with pharmacology as a subject
• The Lego based intervention improves pharmacology knowledge
• The Lego based intervention offers a low-cost option for learner-centred teaching in health professions education institutions

## Notes On Contributors

Professor Brian Kirby BSc(Pharm), PhD, MSc(LMD), PgDip HPE, MPSI is the Deputy Head of School (Programme Innovation) and Associate Professor of Pharmacology in the School of Pharmacy and Biomolecular Sciences, RCSI, Dublin, Ireland. ORCID id: https://orcid.org/0000-0001-9056-830X

Professor Teresa Pawlikowska, BSc, MB, BS, MSc, PhD, MRCP, is the Director of the Health Professions Education Centre, and BEME Coordinator for the BEME International Collaborating Centre, RCSI, Dublin, Ireland.

## Declarations

The author has declared that there are no conflicts of interest.

## Ethics Statement

Ethical approval was given by the Royal College of Surgeons in Ireland Research Ethics Committee (REC1182).

## External Funding

This article has not had any External Funding

## References

[ref1] Aarons L. Foster R. W. Hollingsworth M. Morgan C. H. (1988) Computer-assisted learning lessons in drug disposition and pharmacokinetics. J Pharmacol Methods. 20(2), pp.109–23. 10.1016/0160-5402(88)90071-x 3065577

[ref2] Achike F. I. and Ogle C. W. (2000) Information overload in the teaching of pharmacology. J Clin Pharmacol. 40(2), pp.177–83. 10.1177/00912700022008838 10664924

[ref3] Barry O. P. O’Sullivan E. and McCarthy M. (2015) Periodic review sessions contribute to student learning across the disciplines in Pharmacology. Journal of the Scholarship of Teaching and Learning. 15(1), pp.38–56. 10.14434/josotl.v15i1.12984

[ref4] Beatty S. J. Kelley K. A. Metzger A. H. Bellebaum K. L. (2009) Team-based learning in therapeutics workshop sessions. Am J Pharm Educ. 73(6), p.100. 10.5688/aj7306100 19885069 PMC2769522

[ref5] Bligh D. A. (2000) What’s the use of lectures? 1st edn. San Francisco: Jossey-Bass Publishers.

[ref6] Bruner J. S. and Anglin J. M. (1973) Beyond the Information Given: Studies in the Psychology of Knowing. United States: W W Norton & Company Incorporated.

[ref7] Carpenter J. L. (1995) Cost analysis of objective structured clinical examinations. Acad Med. 70(9), pp.828–33. 10.1097/00001888-199509000-00025 7669163

[ref8] Chang J.-H. and Yeh T.-L. (2015) The Influence of Parent-child Toys and Time of Playing Together on Attachment. Procedia Manufacturing. 3(Supplement C), pp.4921–4926. 10.1016/j.promfg.2015.07.628

[ref9] Coleman I. P. L. Foster R. W. Hollingsworth M. Morgan R. (1995) Drug Targets and Transduction Systems. British Journal of Pharmacology. 115, pp.159P.

[ref10] Das S. Yost S. A. and Krishnan M. (2010) A 10-Year Mechatronics Curriculum Development Initiative: Relevance, Content, and Results-Part I. IEEE Transactions on Education. 53(2), pp.194–201. 10.1109/Te.2008.2011539

[ref11] Dewhurst D. Hughes I. and Williams A. (1996) An interactive computer program to replace in vivo experiments on rat blood pressure for teaching undergraduate students. ATLA - Alternatives to Laboratory Animals. 24, pp.707–714.

[ref12] Dewhurst D. Macleod H. and Norris T. (2000) Independent Student Learning Aided by Computers: An Acceptable Alternative to Lectures? Computers & Education. 35, pp.223–241. 10.1016/S0360-1315(00)00033-6

[ref13] Eberlein T. Kampmeier J. Minderhout V. Moog R. S. (2008) Pedagogies of engagement in science: A comparison of PBL, POGIL, and PLTL. Biochem Mol Biol Educ. 36(4), pp.262–273. 10.1002/bmb.20204 19381266 PMC2665262

[ref14] Ernst H. and Colthorpe K. (2008) Expanding Voluntary Active-learning Opportunities for Pharmacy Students in a Respiratory Physiology Module. American Journal of Pharmaceutical Education. 72(2), p.28. 10.5688/aj720228 18483596 PMC2384203

[ref15] Farne R. (2005) Pedagogy of play. Topoi-an International Review of Philosophy. 24(2), pp.169–181. 10.1007/s11245-005-5053-5

[ref16] Felder R. M. (1993) Reaching the second tier-learning and teaching in college science education. J. Coll. Sci. Teaching. 23, pp.286–290.

[ref17] Frick E. Tardini S. and Cantoni L. (2013) White Paper on LEGO ® SERIOUS PLAY A state of the art of its applications in Europe. Available at: http://www.s-play.eu/attachments/article/70/splay_White_Paper_V2_0_1.pdf( Accessed: 02/10/2017).

[ref18] Gaikwad N. and Tankhiwale S. (2012) Crossword puzzles: self-learning tool in pharmacology. Perspect Med Educ. 1(5-6), pp.237–48. 10.1007/s40037-012-0033-0 23240102 PMC3518804

[ref19] Galvao T. F. Silva M. T. Neiva C. S. Ribeiro L. M. (2014) Problem-Based Learning in Pharmaceutical Education: A Systematic Review and Meta-Analysis. The Scientific World Journal. 2014, p.7. 10.1155/2014/578382 PMC3950357 24701178

[ref20] Hall S. Grant G. Arora D. Karaksha A. (2017) A pilot study assessing the value of 3D printed molecular modelling tools for pharmacy student education. Currents in Pharmacy Teaching and Learning. 9(4), pp.723–728. 10.1016/j.cptl.2017.03.029 29233449

[ref21] Harding S. R. and D’Eon M. F. (2001) Using a Lego-based communications simulation to introduce medical students to patient-centered interviewing. Teach Learn Med. 13(2), pp.130–5. 10.1207/S15328015TLM1302_8 11302033

[ref22] Jungnickel P. W. Kelley K. W. Hammer D. P. Haines S. T. (2009) Addressing competencies for the future in the professional curriculum. Am J Pharm Educ. 73(8), p.156. 10.5688/aj7308156 20221349 PMC2828317

[ref23] Kirkpatrick D. L. and Kirkpatrick J. D. (2007) Implementing the four levels. San Francisco, CA: Berret-Koehler.

[ref24] Kirkpatrick J. D. and Kirkpatrick W. K. (2016) Kirkpatrick’s four levels of training evaluation. 1st edn. Virginia, USA: Association for Talent Development.

[ref25] Krych A. J. March C. N. Bryan R. E. Peake B. J. (2005) Reciprocal peer teaching: students teaching students in the gross anatomy laboratory. Clin Anat. 18(4), pp.296–301. 10.1002/ca.20090 15832347

[ref26] Kuruvilla A. Ramalingam S. Bose A. C. Shastri G. V. (2001) Use of computer assisted learning as an adjuvant to practical pharmacology teaching: advantages and limitations. Indian Journal of Pharmacology. 33, pp.272–275.

[ref27] Letassy N. A. Fugate S. E. Medina M. S. Stroup J. S. (2008) Using team-based learning in an endocrine module taught across two campuses. Am J Pharm Educ. 72(5), p.103. 10.5688/aj7205103 19214257 PMC2630128

[ref28] Lewis D. I. (2014) The pedagogical benefits and pitfalls of virtual tools for teaching and learning laboratory practices in the biological sciences. The Higher Education Academy: STEM.

[ref29] Lewis S. E. (2011) Retention and Reform: An Evaluation of Peer-Led Team Learning. Journal of Chemical Education. 88(6), pp.703–707. 10.1021/ed100689m

[ref30] MacDonald E. and Saarti J. (2006) Beta-blocker blues: pharmacology with a blues beat. Medical Education. 40(11), pp.1127–1128. 10.1111/j.1365-2929.2006.02585.x 17054626

[ref31] McKeachie W. J. (1986) Teaching tips: A guidebook for the beginning college teacher. 8th edn. Lexington, Mass.: D C Heath.

[ref32] Nulty D. D. (2008) The adequacy of response rates to online and paper surveys: what can be done? Assessment & Evaluation in Higher Education. 33(3), pp.301–314. 10.1080/02602930701293231

[ref33] Papert S. and Harel I. (1991) Constructionism.in Constructionism. Westport, CT: Ablex Publishing Corporation.

[ref34] Parkin R. M. (2002) The mechatronics workbench. Computing & Control Engineering Journal. 13(1), pp.16–20. 10.1049/cce:20020103

[ref35] Piaget J. (1951) The Child’s Conception of the World. Lanham: Littlefield Adams Quality Paperbacks.

[ref36] Piaget J. (1971) Psychology and epistemology: Towards a theory of knowledge. (A. Rosin, Trans.). New York: Viking.

[ref37] PLAY L. S. (2002) The Science of LEGO SERIOUS PLAY. Available at: http://www.strategicplay.ca/upload/documents/the-science-of-lego-serious-play.pdf( Accessed: 02/10/2017).

[ref38] Poenaru D. Morales D. Richards A. and O’Connor H. M. (1997) Running an objective structured clinical examination on a shoestring budget. The American journal of surgery. 173(6), pp.538–541. 10.1016/s0002-9610(97)00007-x 9207170

[ref39] Preszler R. W. (2009) Replacing Lecture with Peer-led Workshops Improves Student Learning. CBE Life Sciences Education. 8(3), pp.182–192. 10.1187/cbe.09-01-0002 19723813 PMC2736022

[ref40] Ramsden P. (1991) A performance indicator of teaching quality in higher education: the Course Experience Questionnaire. Studies in Higher Education. 16, pp.129–150. 10.1080/03075079112331382944

[ref41] Reznik V. M. Randolph G. Collins C. M. Peterson B. M. (1993) Cost analysis of dialysis modalities for pediatric acute renal failure. Perit Dial Int. 13(4), pp.311–3, https://www.ncbi.nlm.nih.gov/pubmed/8241334 8241334

[ref42] Richardson A. Bracegirdle L. McLachlan S. I. H. and Chapman S. R. (2013) Use of a Three-Dimensional Virtual Environment to Teach Drug-Receptor Interactions. American Journal of Pharmaceutical Education. 77(1), p.11. 10.5688/ajpe77111 23459131 PMC3578324

[ref43] Richardson A. and Maddock K. (2013) Chairs, bells and students - a novel method to simulate and teach molecular interactions in pharmacology. Pharmacy Education. 13(1), pp.36–39, http://pharmacyeducation.fip.org/pharmacyeducation/article/view/230 (Accessed: 02/10/2017).

[ref44] Roos J. and Victor B. (1999) Towards a new model of strategy-making as serious play. European Management Journal. 17(4), pp.348–355. 10.1016/S0263-2373(99)00015-8

[ref45] Seidl T. (2017) Case Study 1: Playful Team Reflection Using LEGO® Serious Play®. International Journal of Game-Based Learning (IJGBL). 7(3), pp.83–86. 10.4018/IJGBL.2017070108

[ref46] Sharma R. Verma U. Kapoor B. and Chopra V. S. (2004) Novel teaching approaches in Pharmacology. JK Science. 6(3), pp.172–173.

[ref47] Soltis R. Verlinden N. Kruger N. Carroll A. (2015) Process-Oriented Guided Inquiry Learning Strategy Enhances Students. Higher Level Thinking Skills in a Pharmaceutical Sciences Course’, American Journal of Pharmaceutical Education. 79(1), p.11. 10.5688/ajpe79111 PMC4346823 25741027

[ref48] Stevens N. T. McDermott H. Boland F. Pawlikowska T. (2017) A comparative study: do “clickers” increase student engagement in multidisciplinary clinical microbiology teaching? BMC Medical Education. 17(1), p.70. 10.1186/s12909-017-0906-3 28390400 PMC5385002

[ref49] Walpola R. L. Fois R. A. McLachlan A. J. and Chen T. F. (2015) Evaluating the effectiveness of a peer-led education intervention to improve the patient safety attitudes of junior pharmacy students: a cross-sectional study using a latent growth curve modelling approach. BMJ Open. 5(12), p. e010045. 10.1136/bmjopen-2015-010045 PMC4680010 26646830

[ref50] Wilson K. and Korn J. H. (2007) Attention during lectures: Beyond ten minutes. Teaching of Psychology. 34(2), pp.85–89. 10.1080/00986280701291291

[ref51] Wilson K. L. Lizzio A. and Ramsden P. (1997) The development, validation and application of the course experience questionnaire. Studies in Higher Education. 22(1), pp.33–53. 10.1080/03075079712331381121

[ref52] Wilson S. and Becket G. (2012) Using LEGO® SERIOUS PLAY® to explore students’ perceptions of ‘professional’ and ‘unprofessional’ pharmacist identities. International Journal of Pharmacy Practice. 20(Suppl. 1), pp.11–12.

[ref53] Wilson S. B. and Varma-Nelson P. (2016) Small Groups, Significant Impact: A Review of Peer-Led Team Learning Research with Implications for STEM Education Researchers and Faculty. Journal of Chemical Education. 93(10), pp.1686–1702. 10.1021/acs.jchemed.5b00862

[ref54] Yardley S. and Dornan T. (2012) Kirkpatrick’s levels and education ‘evidence’. Med Educ. 46(1), pp.97–106. 10.1111/j.1365-2923.2011.04076.x 22150201

[ref55] Zgheib N. K. Simaan J. A. and Sabra R. (2010) Using team-based learning to teach pharmacology to second year medical students improves student performance. Medical Teacher. 32(2), pp.130–135. 10.3109/01421590903548521 20163228

