# Supplementary material for: Pharmacology through Play: using Lego® to revise core concepts for undergraduates
Source: MedEdPublish (2016). 2019 Nov 14;8:201. [Version 1] doi: 10.15694/mep.2019.000201.1 (PMC10712504; doi:10.15694/mep.2019.000201.1)
Supplement: Peer review history

# Reviewer report: Reviewer response for version 1

**DOI:** 10.21956/mep.19932.r30233

**Published:** 2019-11-22

**Authors:** P Ravi Shankar

**Affiliations:** 1 American International Medical University

This review has been migrated. The reviewer awarded 4 stars out of 5 This is an interesting and well written paper. The authors have used Lego bricks in a group activity to help students learn complex abstract concepts in pharmacology. They have used Lego bricks to explore the topic of agonist receptor interactions. They have also shown two examples of the student creations. If possible, the student groups can explain their models and how it illustrates the particular concept, which will be very interesting. The methodology of the study is well described. The administration of the second set of MCQs immediately after the activity can influence the results. As the authors rightly point out the activity can be expanded to other areas and other groups of students. Peer learning in groups is a powerful learning modality. Here the groups also manipulate Lego bricks to create their own understanding of the concept. The other major advantage is that the activity is relatively cheap compared to more high technology learning options.

Reviewer Expertise:

NA

No decision status is available

# Reviewer report: Reviewer response for version 1

**DOI:** 10.21956/mep.19932.r30237

**Published:** 2019-11-19

**Authors:** Iain Keenan

**Affiliations:** 1 Newcastle University

This review has been migrated. The reviewer awarded 2 stars out of 5 Many thanks to the authors for this interesting, if rather brief, article describing an innovative approach to active learning. The authors have provided a considered introduction outlining some of the supporting evidence and theory behind the approach and an effective discussion setting the findings in the context of current research in this area. The study design requires some imporvements, and a further limitation to consider would be with respect to pre-post testing. The MCQ post-test was identical to the pre-test, so students may have been targeting their learning to those specific questions in the session and this approach may therefore only be testing their short-term memory of those questions. As the authors state, a long-term follow up would also be important. Furthermore, cross-over design is not essential for a valid experimental educational research study. An alternative actvity could be used for the control session (i.e. how the authors would otherwise deliver this session or an alternative active 3D approach, as suggested by another reviewer) for a different but related topic, which is delivered to the entire cohort. This would ensure equitable access for all students). The authors would then compare the difference between both pre and post test scores (i.e. learning performance) for the intervention and control. Additionally, any novel intervention is perhaps likely to provide positive feedback from students. I would like to have seen deeper and richer exploration of the cognitive and constructivist elements and learning value of the approach from the student perspective, perhaps through focus groups and/or semi-structured interviews.

Reviewer Expertise:

NA

No decision status is available

# Reviewer report: Reviewer response for version 1

**DOI:** 10.21956/mep.19932.r30235

**Published:** 2019-11-17

**Authors:** Peter Dieter

**Affiliations:** 1 AMSE

This review has been migrated. The reviewer awarded 2 stars out of 5 The authors examined the student acceptability of a Lego®-based learning session in an attempt to improve pharmacology learning. Most students were positive regarding the session, finding it enjoyable, relevant for their learning and even recommending it be used to explore more complex areas of pharmacology. In addition, there was a significant increase in the MCQ scores following the session. The study used constructivist theory to develop a novel teaching intervention to create a more student-centered, active learning environment. This effective low-cost method could be applied to other teaching programs to enhance student learning.A major concern is that the authors did not compare their method with other methods with respect to effectiveness and costs.

Reviewer Expertise:

NA

No decision status is available

# Reviewer report: Reviewer response for version 1

**DOI:** 10.21956/mep.19932.r30234

**Published:** 2019-11-15

**Authors:** Simonetta Ausoni

**Affiliations:** 1 Department of Biomedical Sciences- University of Padova- School of Medicine

This review has been migrated. The reviewer awarded 3 stars out of 5 This is an interesting paper showing how knowledge in pharmacology can be improved with the application of the Pedagogy of Play. In particular, the authors used Lego as a easy-to-use and cheap method of representation to develop the ability to conceptualize complex issues of pharmacology. Students’ feedback is extremely positive and the assessment shows significant improvement. I think that the paper is an example of good practice and I strongly agree that the teaching of scientific disciplines is very much learner-centered and minimally opened to practice. My major concern is that I do not see the advantages of this approach as compared to other models of representation, such as 3D construction. Although costs of Lego is very low, I am sure that there are many cheap programs that can be used to assist this learning activity. 3D construction could improve molecular shapes, place names to molecular subunits, ions etc., in addition to being computer savable.

Reviewer Expertise:

NA

No decision status is available

# Reviewer report: Reviewer response for version 1

**DOI:** 10.21956/mep.19932.r30236

**Published:** 2019-11-14

**Authors:** Felix Silwimba

**Affiliations:** 1 University of Lusaka

This review has been migrated. The reviewer awarded 5 stars out of 5 this is a clearly written illustrative research report on medical education involving a difficult topic to explain in pharmacology. it has clearly demonstrated use of low cost affordable teaching aid. its methods are applicable everywhere. I recommend the study to medical educators in low-middle income countries.

Reviewer Expertise:

NA

No decision status is available
